# The association between midlife living arrangement and psychiatrist-diagnosed depression in later life: who among your family members reduces the risk of depression?

**DOI:** 10.1038/s41398-022-01880-7

**Published:** 2022-04-11

**Authors:** Kento Ogawa, Kokoro Shirai, Shoko Nozaki, Ryo Shikimoto, Norie Sawada, Masaru Mimura, Hiroyasu Iso, Shoichiro Tsugane, for the Japan Public Health Center-based Prospective Study Group

**Affiliations:** 1 Public Health, Department of Social Medicine, Osaka University Graduate School of Medicine, Suita-shi, Osaka, Japan; 2 Department of Neuropsychiatry, Keio University School of Medicine, Shinjuku-ku, Tokyo, Japan; 3 Epidemiology and Prevention Group, Center for Public Health Sciences, National Cancer Center, Tokyo, Japan

**Keywords:** Depression, Scientific community

## Abstract

This study investigates the longitudinal association between living arrangements and psychiatrists’ diagnosis of depression in the general population. In 1990, 1254 Japanese men and women aged 40–59 years were enroled and completed questionnaires on the living arrangement in the Japan Public Health Center-based Prospective Study (JPHC Study) and participated in a mental health screening (2014–2015). The study diagnosed a major depressive disorder (MDD) assessed by well-trained certified psychiatrists through medical examinations. During the follow-up, a total of 105 participants (36 men and 69 women) aged 64–84 years were diagnosed with MDD by psychiatrists. Living with a child (ren) was associated with a reduced risk of MDD for men but not for women; the respective multivariable ORs (95% CIs) were 0.42 (0.19–0.96) and 0.59 (0.32–1.09). These associations remained unchanged after adjusting for living with spouse and parent(s). In conclusion, living with a child (ren) was associated with a reduced risk of MDD in men, suggesting the role of a child (ren) in the prevention of MDD.

## Introduction

Mental health problems, particularly depression, are already known as serious public health issues. According to WHO reports, depression has emerged as the third leading cause of the global burden of disease in 2004 and is expected to become the first by 2030 [1]. Japan is not an exception [2]. A study reported that the economic burden of depression was approximately $11 billion, with $1570 million relating to direct medical costs, and $2542 million relating to depression-related suicide costs in Japan [3].

There are many potential risk factors for depression. Among them, living arrangements have attracted scientific interest [4, 5] because many countries have undergone rapid demographic changes [6]. The decline in marriage and birth rates has substantially changed living arrangements [6, 7]. These situations are particularly remarkable in Japanese society [7–9].

Previous studies have examined the association between living arrangements and psychological health [10**–**13]. Almost all studies regarding depression were performed in Western countries to examine the association with living arrangements (living alone or not) [10]. Many previous studies in non-Western countries such as China, Singapore, and Thailand used a cross-sectional design or a longitudinal design, but the follow-up duration was as short as one year [14**–**17]. Most studies using a self-report questionnaire used depressive symptoms as an outcome.

We used the data from a large nationwide cohort study with over a 20-year follow-up with certified psychiatrists’ diagnosis of MDD [18**–**20] to investigate the association between the living arrangement of living with a spouse, child(ren), and parent(s) and the risk of MDD among Japanese men and women.

## Methods

### Study population

Participants of the Japan Public Health Center-based prospective study (JPHC study) in 2014–2015, living in the Saku Public Health Center catchment area in Nagano prefecture, were invited for a mental health survey. The JPHC Study was launched at five public health centres (PHCs) in Japan for Cohort I in 1990 [18]. A self-administered questionnaire on demographic information, lifestyle characteristics, and social factors was distributed to uninstitutionalised residents aged 40–59 years in 1990 and follow-ups of 5 years, ten years, and 15 years after the first survey (response rates: 74–81%) were conducted.

**Fig. 1:**
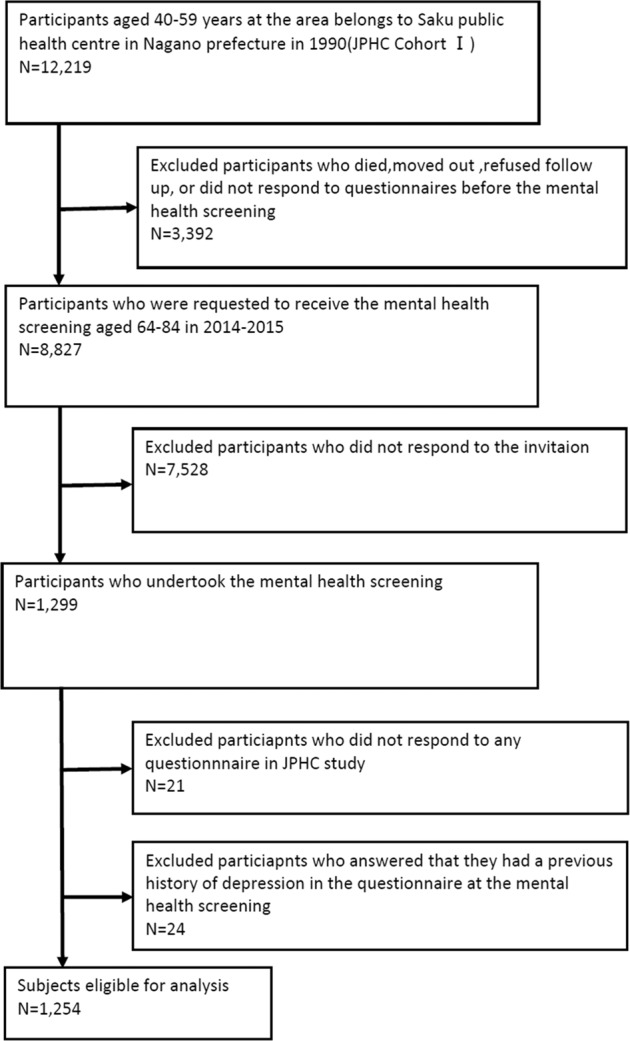
Participants. Flow diagram of the study population selection.

There were 12,219 participants (6172 men and 6047 women) in the baseline survey. After excluding 3392 participants as they moved out of the study area, died, or did not respond to the latter questionnaires during follow-up, we selected the remaining 8827 persons. We invited participants to participate in a mental health survey. A total of 1299 out of 8827 participants (14.7%) responded to the mental health screening. We further excluded 21 participants due to incomplete data for the questionnaires relating to family configuration and 24 participants with a history of depression in the mental health screening questionnaires. The remaining 1254 participants (529 men and 725 women) aged 64–84 were included in the analysis. A flow diagram of the study participants is shown in Fig. 1.

### Assessment of living arrangement

The question about the individual’s family configuration in the baseline questionnaire was, “Are you living with someone (spouse, child (ren), parent(s), others, alone) together now?” According to the Japanese culture, “others” are regarded as other family members; siblings, grandparents, uncles, aunts, cousins, in-laws, etc. The same question was repeated for each follow-up survey. In the present study, we used a questionnaire of 1990, and only 15 persons (five men and ten women) living alone participated; hence, we could not analyse them as explanatory variables.

### Covariates

Regarding past histories, we followed up the participants from 1990 until the screening in 2014–2015 and registered participants’ conditions, including survival and medical status, within the catchment areas of Saku City. We assessed the incidence of cancer using medical records from each hospital in the study area. We interviewed past histories of depression, diabetes mellitus, stroke, and myocardial infarction by self-administrated questionnaire of this mental health survey. All other covariates, except for past histories (smoking status, alcohol frequency, sleeping duration, and occupation), were questioned through the baseline survey in 1990.

### Confirmation of depression

Certified psychiatrists assessed all participants in this mental health screening. First, we administrated the Center for Epidemiological Scale-Depression (CES-D) [21, 22] and the Patient Health Questionnaire-9 (PHQ-9) [23, 24] screening tests at the same time. Second, well-trained board-certified psychiatrists interviewed the participants by referring to the CES-D and PHQ-9 scores. Finally, the psychiatrists assessed whether the participant was diagnosed with MDD based on the Diagnostic and Statistical Manual of Mental Disorders, 4th Edition (DSM-IV) criteria [19, 20] and reached a consensus for final diagnosis when each psychiatrist’s diagnosis was different.

If a patient experiences depressive symptoms, they are not always diagnosed with depression [25]. Mild cognitive impairment (MCI), dementia, and pseudodementia may be associated with similar symptoms [25**–**27]. It is often difficult for general doctors to distinguish MDD from others, and patients sometimes have overlapping diseases [25, 28]. Well-trained board-certified psychiatrists met the 1299 participants, confirmed their self-report questionnaires, and assessed whether the participants currently met the DSM-IV criteria for MDD after considering whether their depressive symptoms caused clinically significant distress or impairment [19].

### Statistical analysis

Logistic regression analyses were performed to calculate the odds ratios (ORs) and 95% confidence intervals (CIs) of MDD associated with the family configuration. We adjusted for age (years, continuous) and sex in the first model and adjusted for other lifestyle-related factors and health history in the second model. These factors included smoking status (never, former, current), alcohol frequency (seldom, 1–3 times per month, 1–2 times per week, 3–4 times per week, 5–6 times per week, every day), sleeping duration (≤4 h, 5–9 h, ≥10 h), occupation (professional, managerial, white-collar, and blue-collar jobs), and education (primary education, lower secondary education, upper secondary education, post-secondary education), history of cancer (yes or no), stroke (yes or no), myocardial infarction (yes or no), and diabetes mellitus (yes or no). The rate of missing values was lower than 0.2–1.4%. Missing data were assumed to be missing at random (MAR), and we used multiple imputations to handle missing data of confounding variables using the ‘mice’ package in R software. All variables in the dataset used in this study were included in the imputation model. Results across five imputed datasets were combined by averaging, and standard errors were adjusted to reflect both within-imputation variability and between-imputation variability in the pooling phase [29]. The level of statistical significance was set α = 0.05 (two-tailed). All statistical analyses were conducted by the R software (version 3.5.3; https://www.r-project.org/).

## Results

The characteristics of study participants who had lived with a child (ren), a spouse, and a parent(s) at baseline are shown in Table 1. The median age of the patients was 47 years, and 58% were women. Except for age, no significant differences were found for any other characteristics. Among the 1254 participants (529 men and 725 women), 105 participants (36 men and 69 women) were diagnosed with MDD by board-certified psychiatrists.

**Table 1:** Characteristics of participants according to living arrangement.

	Not living with spouse	Living with spouse	Not living with child(ren)	Living with child(ren)	Not living with parent(s)	Living with parent(s)
Men						
*N*	37	492	92	437	265	264
Information at screening						
Age at baseline	46 [42, 53]	48 [43, 52]	53.5 [47, 56]	47 [42, 51]	49 [45, 54]	46 [42, 51]
History of cancer, % (*n*)	21.6 (8)	15.7 (77)	16.3 (15)	16.0 (70)	16.6 (44)	15.5 (41)
History of myocardial infarction, % (*n*)	2.7 (1)	3.0 (15)	1.1 (1)	3.4 (15)	2.6 (7)	3.4 (9)
History of stroke, % (*n*)	5.4 (2)	5.1 (25)	6.5 (6)	4.8 (21)	6.4 (17)	3.8 (10)
History of diabetes, % (*n*)	0 (0)	0.4 (2)	1.1 (1)	0.2 (1)	0.8 (2)	0 (0)
Information from baseline survey						
Current smoker, % (*n*)	66.7 (24)	45.2 (222)	46.7 (43)	46.7 (203)	47.0 (124)	46.4 (122)
Regular drinker, % (*n*)	43.2 (16)	40.7 (200)	38.0 (35)	41.4 (181)	38.9 (103)	42.8 (113)
Post-secondary education, % (*n*)	10.8 (4)	20.7 (100)	18.9 (17)	20.3 (87)	20.1 (52)	20.1 (52)
Blue collar job, % (*n*)	62.2 (23)	55.8 (273)	52.7 (48)	57.0 (248)	54.4 (143)	58.2 (153)
White collar job (service), % (*n*)	13.5 (5)	11.7 (57)	13.2 (12)	11.5 (50)	14.4 (38)	9.1 (24)
White collar job (office), % (*n*)	13.5 (5)	20.4 (100)	20.9 (19)	19.8 (86)	19.0 (50)	20.9 (55)
Professional and technical personnel and Managerial, % (*n*)	10.8 (4)	11.7 (57)	12.1 (11)	11.5 (50)	11.4 (30)	11.8 (31)
Other job, % (*n*)	0 (0)	0.4 (2)	1.1 (1)	0.2 (1)	0.8 (2)	0 (0)
Short and long sleeper, % (*n*)	0 (0)	1.6 (8)	0 (0)	1.8 (8)	0.4 (1)	2.7 (7)
Women						
*N*	116	609	138	587	427	298
Information at screening						
Age at baseline	47 [43, 52]	48 [43, 52]	52 [49, 55]	46 [42, 51]	49 [44, 53]	46 [42, 50]
History of cancer, % (*n*)	14.7 (17)	11.0 (67)	15.9 (22)	10.6 (62)	13.6 (58)	8.7 (26)
History of myocardial infarction, % (*n*)	1.7 (2)	0.7 (4)	0 (0)	1.0 (6)	0.9 (4)	0.7 (2)
History of stroke, % (*n*)	1.7 (2)	3.1 (19)	2.9 (4)	2.9 (17)	3.0 (13)	2.7 (8)
History of diabetes, % (*n*)	1.7 (2)	0.5 (3)	1.4 (2)	0.5 (3)	0.7 (3)	0.7 (2)
Information from baseline survey						
Current smoker, % (*n*)	6.9 (8)	3.1 (19)	2.2 (3)	4.1 (24)	4.5 (19)	2.7 (8)
Regular drinker, % (*n*)	1.7 (2)	2.6 (16)	4.3 (6)	2.1 (12)	3.5 (15)	1.0 (3)
Post-secondary education, % (*n*)	16.5 (19)	14.4 (86)	16.3 (22)	14.4 (83)	16.2 (68)	12.7 (37)
Blue collar job, % (*n*)	56.0 (65)	51.3 (309)	50.7 (70)	52.4 (304)	50.2 (212)	54.7 (162)
White collar job(service), % (*n*)	11.2 (13)	16.1 (97)	21.0 (29)	14.0 (81)	17.3 (73)	12.5 (37)
White collar job(office), % (*n*)	15.5 (18)	13.6 (82)	8.7 (12)	15.2 (88)	13.5 (57)	14.5 (43)
Professional and technical personnel and Managerial, % (*n*)	9.5 (11)	9.5 (57)	11.6 (16)	9.0 (52)	8.8 (37)	10.5 (31)
Other job, % (*n*)	7.8 (9)	9.5 (57)	8.0 (11)	9.5 (55)	10.2 (43)	7.8 (23)
Short and long sleeper, % (*n*)	0.9 (1)	0.7 (4)	2.2 (3)	0.3 (2)	0.7 (3)	0.7 (2)

Age: median [lower, and upper quantiles].

Regular drinker means a person who drinks alcohol beverage every day.

Table 2 shows the odds ratios (ORs) of MDD according to living arrangements. Among the group living with a child (ren) or not, ORs (95% CI) of MDD were 0.42 (0.19 to 0.96) for men, 0.59 (0.32 to 1.09) for women and 0.53 (0.32 to 0.85) for total participants. When we tested our hypothesis of no association with Bonferroni correction in total participants, the *P* value was 0.012, which was under 0.0167(=0.05/3), rejecting the hypothesis. These associations remained unchanged after further adjustment for living with a spouse and living with parent(s).

**Table 2:** Odds ratios and 95% confidence intervals for depression according to living arrangement.

	Not living with spouse	Living with spouse	Not living with child(ren)	Living with child(ren)	Not living with parent(s)	Living with parent(s)
Men						
No. of cases/ *N*	3/37	33/492	10/92	26/437	17/265	19/264
Age-adjusted OR	1	0.83 (0.24–2.83)	1	0.45 (0.20–1.00)	1	1.09 (0.55–2.17)
Multivariable OR (95% CI)^1^	1	0.87 (0.25–3.25)	1	0.42 (0.19–0.96)	1	1.10 (0.54–2.23)
Multivariable OR (95% CI)^2^	1	1.52 (0.36–6.37)	1	0.38 (0.15–0.92)	1	1.17 (0.57–2.39)
Women						
No. of cases/ *N*	14/116	55/609	22/138	47/587	43/427	26/298
Age-adjusted OR	1	0.67 (0.36–1.26)	1	0.62 (0.35–1.10)	1	1.04 (0.62–1.77)
Multivariable OR (95% CI)^1^	1	0.63 (0.32–1.22)	1	0.59 (0.32–1.09)	1	1.20 (0.69–2.08)
Multivariable OR (95% CI)^2^	1	0.62 (0.32–1.20)	1	0.58 (0.31–1.07)	1	1.27 (0.72–2.20)
Total						
No. of cases/ *N*	17/153	88/1101	32/230	73/1024	60/692	45/562
Age-and-sex-adjusted OR	1	0.72 (0.41–1.25)	1	0.55 (0.34–0.89)	1	1.06 (0.70–1.60)
Multivariable OR (95% CI)^1^	1	0.69 (0.39–1.22)	1	0.53 (0.32–0.85)	1	1.06 (0.70–1.63)
Multivariable OR (95% CI)^2^	1	0.73 (0.41–1.30)	1	0.53 (0.33–0.87)	1	1.13 (0.73–1.73)

^1^Adjusted further for smoking status, drinking status, educational status, occupational status, sleep duration, past history of cancer, stroke, miocardial infarction and diabetes melitus.

^2^Adjusted further for living with child and living with parent for the spouse analysis, living with spouse and living with parent for the child analysis, living with child and living with spouse for the parent analysis.

Fifteen persons living alone participated in the present study. However, we found that the association between living with a child (ren) and the risk of MDD remained unchanged even after excluding participants living alone (Supplementary Table 1). We conducted another sensitivity analysis, dividing the group of ‘not living with a child(ren)’ into two groups of ‘not having a child(ren)’ and ‘having but not living with a child(ren)’ because the child(ren) of participants had already moved out from home at the baseline survey. Then, we examined the ORs of MDD in ‘having but not living with a child(ren)’ and ‘living with a child(ren)’ in reference to ‘not having a child(ren),’ and found that these two groups had a lower risk of MDD in total participants (Supplementary Table 2).

However, no significant associations were observed between living with a spouse, parent(s), and risk of MDD.

## Discussion

In this prospective study of Japanese men and women aged 40–59 years, we found that living with a child (ren) was associated with a 62% lower risk of MDD after adjusting for potential confounding factors for men, and a weak and insignificant association was observed for women.

A cross-sectional study of 1561 parents aged ≥60 years in the Anhui Province, China, found that older adults living with three generations or living with grandchildren had a lower prevalence of depressive symptoms than those living in single-generation households [14]. Another cross-sectional analysis under the China Health and Retirement Longitudinal Study (CHARLS) of 6001 men and women aged ≥60 years showed that older adults living with both a child and spouse or living alone were more likely to have depressive symptoms than those living only with a spouse (odds ratio = 1.23 [1.06–1.42] and 1.40 [1.03–1.92], respectively) [17]. These two studies showed the opposite effect of living with descendants. However, the cross-sectional design was liable to reverse causation because family members likely supported those who experienced depression episodes in their families [30].

Moreover, a 3-year follow-up of the Japan Gerontological Evaluation Study (JAGES) of 19,656 men and 22,513 women aged ≥65 years reported that living with a spouse and child for men was associated with increased odds of depressive symptoms (odds ratio = 1.19 [1.04–1.36]) In the 65–74 years group, but the odds reduced for women (odds ratio = 0.81 [0.71–0.94]) [10]. In the present study we found a strong association between living with a child (ren) and a reduced risk of MDD in men but not in women. This difference may be due to the different durations of follow-up and outcome estimation. While JAGES used depressive symptoms assessed by the self-report questionnaire, this study used a certified psychiatrist’s diagnosis.

The absence of a significant association between living with a spouse and the risk of MDD in the present study could be due to the small number of persons who did not live with their spouse. A cross-sectional study of 4685 Finnish men and women aged 30–64 years showed that persons living alone were more likely to have depressive disorders than persons living with spouses [13]; ORs (95% CI) of MDD were 1.62 (1.01–2.62) for men and 1.66 (1.12–2.45) for women. We found no association between living with parents and the risk of MDD. To the best of our knowledge, no studies have investigated the effect of living with parent(s) separately on the risk of MDD.

Almost all of the previous studies conducted in Western countries have not focused on who people live with but on whether people live alone or not [10]. A past systematic review of 74 community-based mental health surveys on depression reported that living alone was identified as a risk factor for depression in aged populations in the world including Europe, North America, South America, Australia, and Asia [31].

Living with a child (ren) may soothe men’s stress, probably because they can receive social and financial support. According to the Annual Report on Health, Labour and Welfare 2015–2016, the first leading cause of worry in old age among Japanese was a healthy life, and the second was finance [32]. More than 72 percent of Japanese aged ≥40 years wanted to stay in their own houses instead of the nursing home when they were older and tended to seek social support from their child(ren) [32]. More than 60 per cent of persons aged ≥70 years wanted to live with a child (ren) or nearby [32]. Additionally, 78 per cent of old-age households depend mainly on public pension income [33]. However, the population census in Japan has shown that the proportion of people living alone or residing with aging parents has increased for both middle-aged women and married couples since the 1970s [34].

An advantage of the present study is that we used long-term follow-up data instead of cross-sectional data. We could reduce the possibility of reverse causality, a limitation for most cross-sectional previous research. Another strength of this study is that we minimised the outcome measurement errors by the board-certified psychiatrists’ diagnosis, which is highly recommended [28].

Several limitations of our study warrant discussion. First, the statistical power was insufficient to examine the association, as the limited number of persons living alone enroled. Second, selection bias may have occurred because 14% of the eligible participants participated in this study. Last, there was residual or uncontrolled confounding for the association between living arrangements and risk of MDD such as household income, a family history of mental disease and depression/psychiatric status.

In conclusion, our study indicated that living with a child (ren) was associated with a reduced risk of MDD. Our results suggest that elderly persons may benefit by preventing MDD, possibly through social and financial support from children.

## Supplementary information

Supplemental Table 1.

Supplemental Table 2.

## Data Availability

For information on how to apply to gain access to JPHC data, follow the instructions at https://epi.ncc.go.jp/en/jphc/805/8155.html.
