# Supplementary figures and images for: The association between midlife living arrangement and psychiatrist-diagnosed depression in later life: who among your family members reduces the risk of depression?
Source: Transl Psychiatry. 2022 Apr 11;12:156. doi: 10.1038/s41398-022-01880-7 (PMC9001692; doi:10.1038/s41398-022-01880-7)

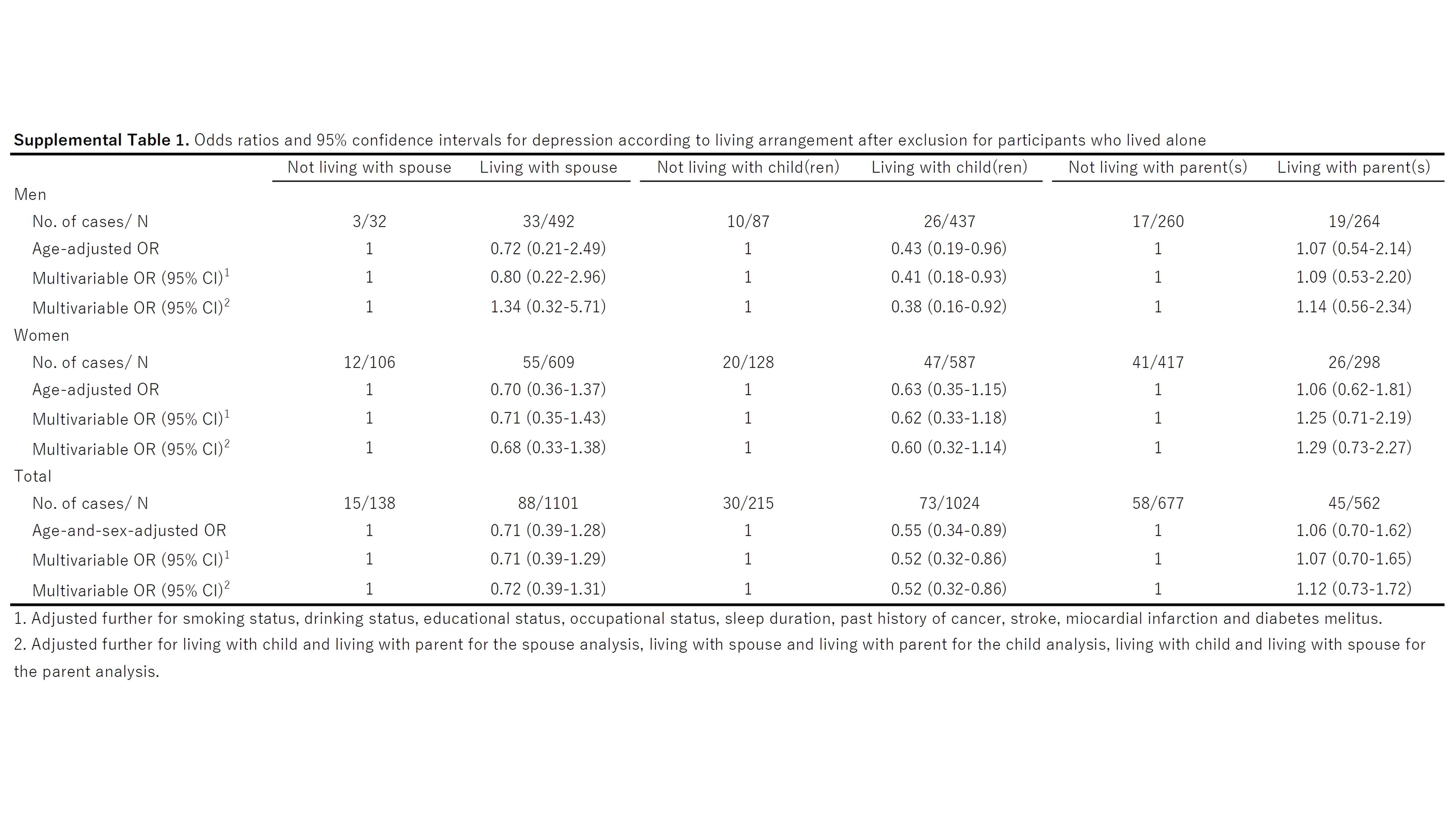

Supplement: Supplementary file 1 — Supplemental Table 1. [file 41398_2022_1880_MOESM1_ESM.tif]

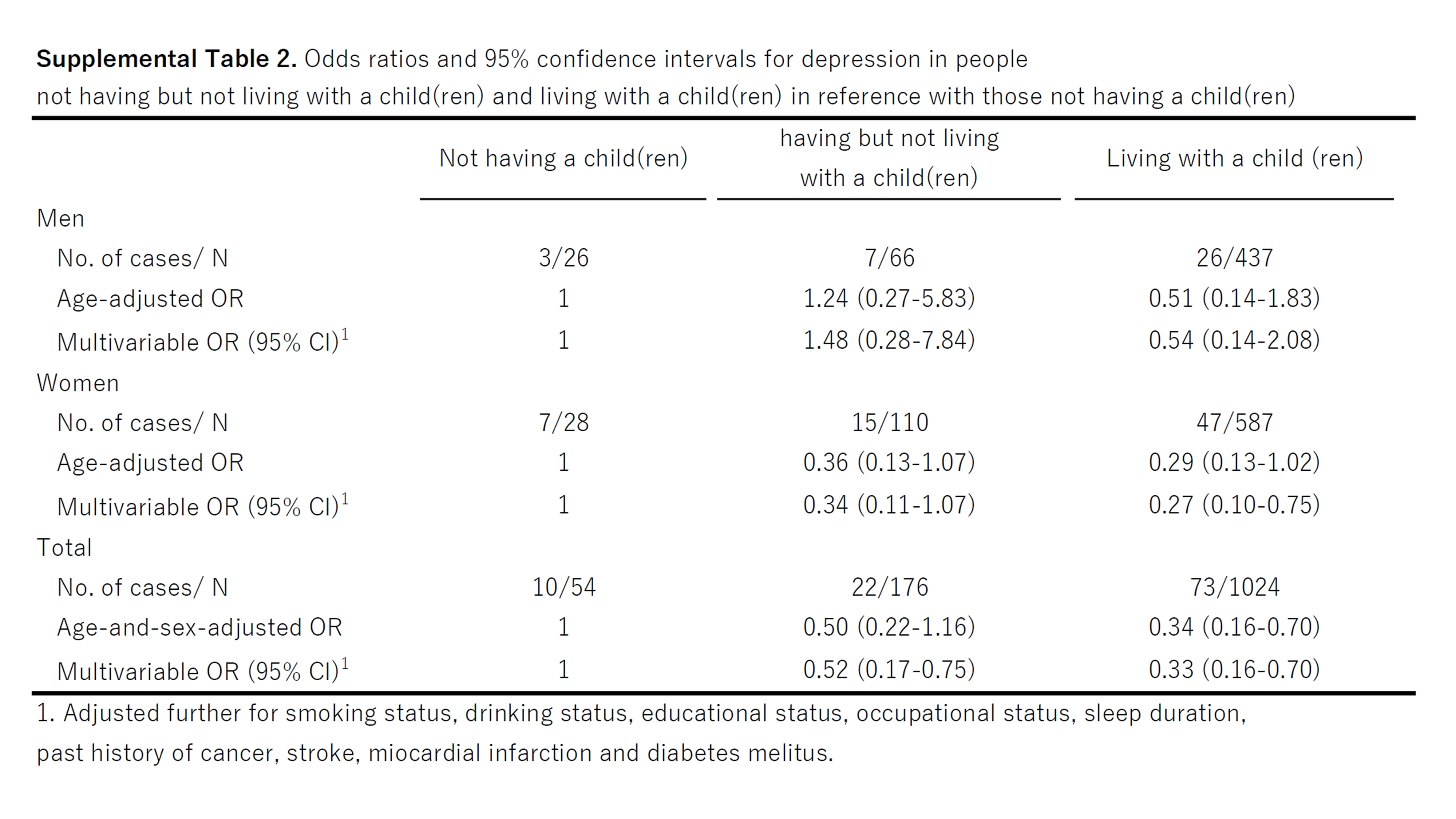

Supplement: Supplementary file 2 — Supplemental Table 2. [file 41398_2022_1880_MOESM2_ESM.tif]
